# Scope, Breadth, and Differences in Online Physician Ratings Related to Geography, Specialty, and Year: Observational Retrospective Study

**DOI:** 10.2196/jmir.7475

**Published:** 2018-03-07

**Authors:** Jessica Janine Liu, John Justin Matelski, Chaim M Bell

**Affiliations:** ^1^ Department of Medicine University of Toronto University Health Network Toronto, ON Canada; ^2^ Biostatistics Research Unit University Health Network Toronto, ON Canada; ^3^ Sinai Health System Department of Medicine University of Toronto Toronto, ON Canada

**Keywords:** quality improvement, patient satisfaction, patient-centered care, online ratings

## Abstract

**Background:**

Physician ratings websites have emerged as a novel forum for consumers to comment on their health care experiences. Little is known about such ratings in Canada.

**Objective:**

We investigated the scope and trends for specialty, geographic region, and time for online physician ratings in Canada using a national data source from the country’s leading physician-rating website.

**Methods:**

This observational retrospective study used online ratings data from Canadian physicians (January 2005-September 2013; N=640,603). For specialty, province, and year of rating, we assessed whether physicians were likely to be rated favorably by using the proportion of ratings greater than the overall median rating.

**Results:**

In total, 57,412 unique physicians had 640,603 individual ratings. Overall, ratings were positive (mean 3.9, SD 1.3). On average, each physician had 11.2 (SD 10.1) ratings. By comparing specialties with Canadian Institute of Health Information physician population numbers over our study period, we inferred that certain specialties (obstetrics and gynecology, family practice, surgery, and dermatology) were more commonly rated, whereas others (pathology, radiology, genetics, and anesthesia) were less represented. Ratings varied by specialty; cardiac surgery, nephrology, genetics, and radiology were more likely to be rated in the top 50th percentile, whereas addiction medicine, dermatology, neurology, and psychiatry were more often rated in the lower 50th percentile of ratings. Regarding geographic practice location, ratings were more likely to be favorable for physicians practicing in eastern provinces compared with western and central Canada. Regarding year, the absolute number of ratings peaked in 2007 before stabilizing and decreasing by 2013. Moreover, ratings were most likely to be positive in 2007 and again in 2013.

**Conclusions:**

Physician-rating websites are a relatively novel source of provider-level patient satisfaction and are a valuable source of the patient experience. It is important to understand the breadth and scope of such ratings, particularly regarding specialty, geographic practice location, and changes over time.

## Introduction

Patients’ abilities to discern health care quality are often underappreciated, despite evidence that low patient satisfaction scores and complaints against physicians are linked to increased risk management episodes, malpractice lawsuits, readmission rates, and even increased mortality for selected diagnoses [1-5]. Over the last decade, physician-rating websites have become a popular source of patient satisfaction data [6]. Such websites represent unsolicited reflections of the patient experience with their physicians in comparison to more traditional methods such as surveys. In the United States, the use of physician-rating websites is rapidly increasing, whereas other countries have reported more moderate growth [6,7]. In addition to private online physician websites, government or health insurer-developed sites are also being used in countries such as the United Kingdom and Germany [8,9]. Together, these physician-rating websites may impact patient health care decision making, as data suggests approximately one-third of users have searched for physicians online and report making decisions regarding physician selection based on these ratings [10]. Online physician-rating websites may also impact physician behaviors; over the last five years, physicians have been increasingly responding online to their ratings [11]. Hence, this data source may have significant implications on health care practice and behavior.

Most previous work on online physician ratings has focused on reviewing the frequency and usage among different physician specialties in the United States, China, and Germany [6,12-26], as well as exploring awareness and perceptions among physicians and consumers [10,11,27-29]. More recently, the focus has been to correlate online ratings with quality outcomes or surrogates such as postoperative mortality and surgical volumes with variable findings, depending on the quality outcome in question [6,30-38]. It has been estimated that one in six physicians are rated online, and most ratings are positive [6,12-4,17-19,28]. Although the use of physician-rating websites is increasing overall [6,7,39], for frequency of ratings, US studies have reported that the mean number of ratings per physician is low overall, ranging from two to four ratings per physician [6,17,21]. Several studies have focused on differences in ratings according to specialties [6,14,20-22]. Certain types of physicians, such as obstetricians, dermatologists, surgeons, and family physicians, are more frequently rated than other specialists. Board-certified, younger physicians have been shown to be rated more favorably than non-board-certified, younger physicians [6,14]. Other studies have investigated the relationship between practice location (such as city size) and online ratings. In the United States, physicians in the southern states had a higher likelihood of positive ratings than other parts of the country, whereas others have shown no difference in ratings with respect to practice location and city size [6,20-22].

In Canada, there is currently little information available on the use of physician-rating websites. Our study sought to investigate the nature and trends of online physician ratings in Canada over a nearly 8-year period. The goals of this study were to (1) determine whether online ratings for physicians differed depending on physician specialty, (2) investigate whether physician practice location affected online ratings, and (3) examine possible trends in ratings over time by year of rating. We also compared the number and frequency of ratings by specialty to determine whether certain specialties were rated online more frequently than expected based on their representation in the overall physician population. Based on previous studies, we hypothesized that certain specialties, such as obstetrics and family medicine, would be rated more frequently than others, such as pathology or radiology. We also felt that the quality of ratings would be positive overall and that differences in ratings would exist across specialties and geographic practice location. We suspected that there would be no differences in quality of ratings over time, but that the absolute number of ratings would be steadily increasing over our study period.

## Methods

### Overview

We accessed a national database of all Canadian physicians rated from January 2005 to September 2013 (N=640,603 ratings) [40]. RateMDs was founded in the United States in 2004 and is currently among the most popular physician-rating websites in Canada and the United States by user traffic [13,18]. No registration or subscription is required to view or post a rating, and there are no incentives to rate a physician. Physicians are rated on a scale of 1 to 5 (described by the website as 1=“terrible,” 2=“poor,” 3=“okay,” 4=“good,” 5=“excellent”). Ratings were given for each of the following domains: staff, punctuality, helpfulness, and knowledge. A mean overall score is posted for each physician. Physician profiles are created or searched for by the rater, and users provide ratings and may provide free-text comments if desired. Our dataset included deidentified data for 57,412 physicians, including specialty, practice region (city and province), date of rating, and scores on each of four domains, from which we calculated an average cumulative rating for each physician. This dataset included all physicians in Canada who were rated on RateMDs during our study period.

Mean number of ratings and mean ratings were calculated for all physicians, each website specialty, and province. To compare the relative proportions of physicians by specialty, we grouped specialties according to Canadian Institute of Health Information (CIHI) categories [41]. We considered “obstetrics and gynecology” as distinct from “surgery” because previous research demonstrated high numbers of ratings for this group [6,14]. We calculated each physician specialty’s online presence by grouping online specialties into CIHI specialty categorizations and divided the number of physicians rated online for that specialty by the total number of physicians in the online database. We then calculated and compared these values to the mean annual number of physicians divided by the total annual physician population for CIHI specialties from 2005 to 2013 (to match our online ratings data period). This allowed us to infer whether a specialty was rated more or less frequently than expected based on the mean annual physician population for that specialty.

### Statistics

For statistical analysis, our objective was to recognize and compare differences in favorable versus unfavorable ratings for physician specialty, geographic practice location, and year of rating. We constructed a binary variable indicating whether each rating was greater than, less than, or equal to the median rating, which was 4.5 out of 5. We thus considered whether ratings were in the top 50th percentile of all ratings for one of three predictors: physician specialty, province, and year. For each level of predictor, the proportion of ratings greater than 4.5 was reported with a 95% confidence interval and a *P* value against the null hypothesis that the true proportion was equal to 0.5. In this way, we were able to stratify specialties, practice location by province, and year of rating according to likelihood of positive ratings. All analyses were performed using *prop.test* in R version 3.0.2.

### Ethics

When submitting research ethics board approval, we were informed that the requirement for ethics approval was waived because data were publicly available.

## Results

### Findings

From February 2005 to September 2013, there were 640,603 ratings for 57,412 unique physicians. Ratings were generally positive (mean 3.9, SD 1.3). Using the online rating website’s rating descriptions, this translated to a mean rating that fell between “okay” and “good.” The mean number of ratings per physician was 11.2 (SD 10.1; see Table 1). During our study period, the mean annual number of total physicians in Canada was 66,026.1 (SD 5748.2). The largest group of physicians, by medical specialty, was family medicine/general practice (n=30,818 physicians). This group had 370,972 unique ratings and, on average, had 12 ratings per physician, with a mean overall rating of 3.9 (SD 1.3). Internal medicine (including its subspecialties) accounted for 53,818 total ratings of 6677 individual physicians, with 8.1 ratings per physician (SD 7.7) and a mean rating of 3.98 (SD 1.31) out of 5. Surgery (including its subspecialties) included 22,811 total ratings of 2472 individual physicians, with 11.9 ratings per physician (SD 10.7) and an overall mean rating of 4.01 (SD 1.32) out of 5. We found that certain specialties had relatively increased numbers of per-physician ratings, including reproductive endocrinology (mean 19.7, SD 15.2), cosmetics/plastic surgery (mean 16.7, SD 16.1), and obstetrics and gynecology (mean 17.6, SD 16.1). Additionally, certain medical specialties had lower numbers of rated physicians as well as per-physician ratings, including radiologists (total number of rated physicians: 330, mean per-physician ratings 3.0, SD 2.8), pathologists (total number of rated physicians: 13, mean per-physician ratings 4.4, SD 8.1), and medical geneticists (total number of rated physicians: 26, mean per-physician ratings 2.6, SD 4.9; see Table 1).

### Differences in Frequencies of Ratings According to Specialty

For each specialty, we calculated the percentage of physicians with online ratings divided by the total online physician population, and compared it to the percentage of physicians in a given specialty divided by the total annual physician populations for CIHI specialties. Certain specialties were more frequently rated than expected based on their proportion in the national population, notably obstetrics and gynecology (4.3% of online cohort vs 2.5% of mean total annual obstetrics and gynecology population), dermatology (1.2% vs 0.8%), family practice (53.7% vs 45.2%), internal medicine (including its subspecialties; 12.0% vs 10.3%), emergency medicine/critical care (2.4% vs 1.1%), and surgery (14.3% vs 10.0%), whereas others were less represented, including anesthesia (1.4% vs 4.1%), radiology (0.6% vs 3.3%), psychiatry (5.0% vs 6.3%), and pathology (<0.01% vs 1.9%; see Table 1).

### Differences in Quality of Ratings for Physician Specialty

**Figure 1:**
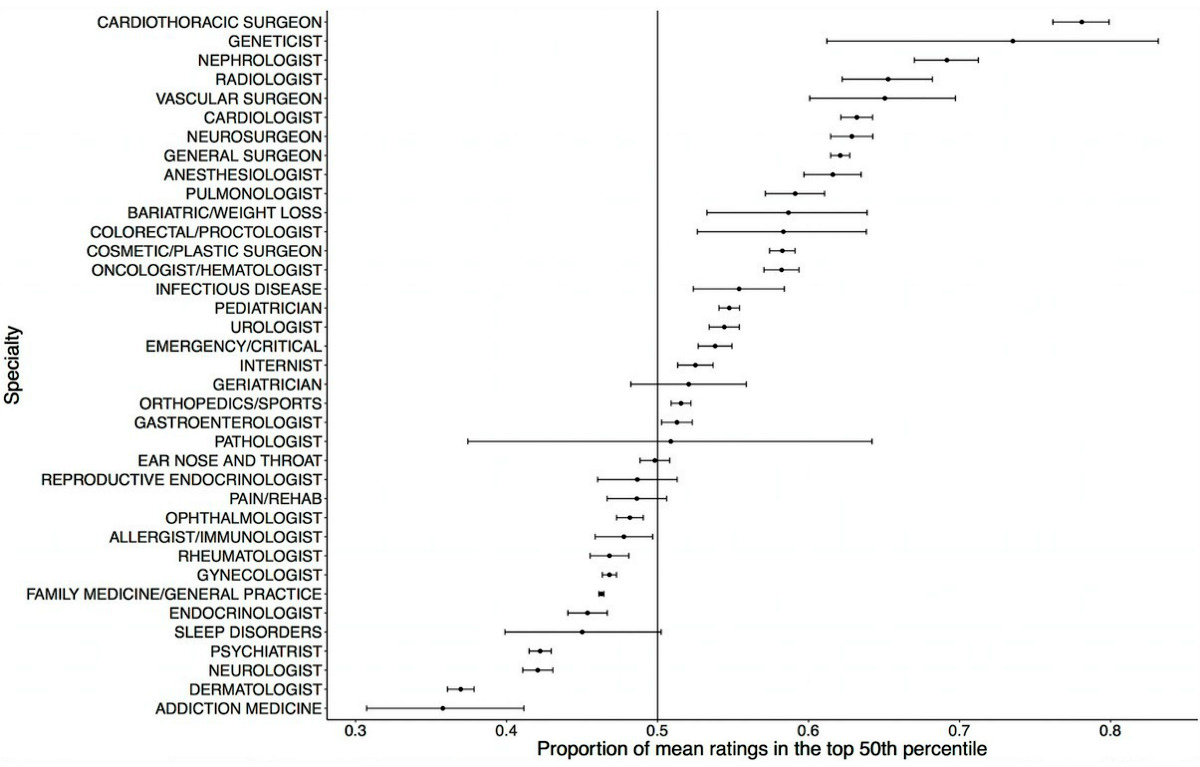
Proportion of mean ratings, by specialty, in the top 50th percentile of all rated physicians (2005-2013) with 95% confidence intervals depicted for each proportion.

We investigated whether there were differences in the quality of ratings depending on physician specialty. We found that ratings for certain specialties were more likely to be in the top 50th percentile of all ratings, including cardiac surgery (probability of a rating greater than the median of 4.5 was 78.1%, *P*<.001), genetics (73.5%, *P*<.001), nephrology (69.2%, *P*<.001), radiology (65.3%, *P*<.001), and vascular surgery (65.1%, *P*<.001). The bottom four physician specialties included psychiatry (42.2%, *P*<.001), neurology (42.1%, *P*<.001), dermatology (37.0%, *P*<.001), and addiction medicine (35.8%, *P*<.001; Figure 1; Multimedia Appendix 1). Family medicine/general practice comprised our largest group of physicians in the online cohort, as well as one of the largest groups of physicians represented in the mean annual physician population. Regarding likelihood of a favorable rating, family medicine/general practice was among the bottom seven physician specialties (46.3%, *P*<.001; Figure 1; Multimedia Appendix 1).

### Differences in Frequency of Ratings for Physician Practice Location (by Province)

We found that Ontario had both the highest number of ratings and the highest number of rated unique physicians (244,635 ratings for 20,740 physicians), followed by Quebec (116, 041 for 13,460 physicians), then British Columbia (101,152 ratings for 8398 physicians). The lowest number of ratings for the lowest number of physicians was found in the less densely populated regions of the Northwest Territories/Yukon/Nunavut (802 ratings for 126 physicians) and Prince Edward Island (2534 ratings for 242 physicians).

For most provinces, per-physician number of ratings ranged from 10 to 13, with the exception of Quebec and the Northwest Territories/Yukon/Nunavut (ratings per physician 8.62 and 6.37, respectively).

### Differences in Quality of Ratings for Physician Practice Location (by Province)

We also found differences in a physician’s likelihood of a positive rating depending on practice location. Broadly speaking, physicians who practiced medicine in the eastern geographic locations of the country had a higher likelihood of being favorably rated than those who practiced in central or western Canada.

**Table 1:** Number of ratings, unique number of physicians, and descriptive statistics and relative proportions of rated physicians grouped by Canada Institute of Health Information (CIHI) specialty (2005-2013).

Medical specialty				Ratings, n		Unique rated physicians, n		Ratings per physician, mean (SD)		Overall rating, mean (SD)		Annual physician population^a^ (%), mean (SD)		% of online cohort		% of mean annual physician population^b^
Family medicine				370,972		30,818		12.0 (9.7)		3.9 (1.3)		33,180.0 (4745.8)		53.7		45.2
**Internal medicine**				53,818		6677		8.1 (7.7)		4.0 (1.3)		7528.1 (778.3)		12.0		10.3
		Allergy/immunologist	2690		235		11.5 (9.6)		3.8 (1.3)							
		Cardiologist	8192		1278		6.4 (5.8)		4.20 (1.2)							
		Colorectal/proctologist	312		36		8.7 (9.5)		4.02 (1.4)							
		Gastroenterologist	9395		854		11.0 (8.8)		3.91 (1.3)							
		Endocrinologist	5670		563		10.1 (8.6)		3.81 (1.3)							
		Reproductive endocrinologist	1418		72		19.7 (15.2)		3.91 (1.3)							
		Geriatrician	678		168		4.0 (4.4)		3.84 (1.4)							
		Infectious disease	1074		199		5.4 (6.1)		4.04 (1.3)							
		Internist	7045		1112		6.3 (6.4)		3.90 (1.4)							
		Nephrologist	1868		364		5.1 (4.4)		4.36 (1.1)							
		Oncology/hematologist	7038		1086		6.5 (6.1)		4.12 (1.2)							
		Pulmonologist	2463		383		6.4 (5.8)		4.10 (1.3)							
		Rheumatologist	5915		472		12.5 (9.4)		3.81 (1.4)							
		Sleep disorders	371		57		6.5 (6.9)		3.7 (1.4)							
Anesthesia				2589		622		4.16 (5.0)		4.14 (1.3)		2713.1 (287.0)		1.1		4.1
Obstetrics and gynecology				43,627		2472		17.6 (15.0)		3.88 (1.3)		1709.0 (292.3)		4.3		2.5
**Surgery**				98,045		8235		11.9 (10.7)		4.01 (1.3)		6618.2 (375.3)		14.3		10.0
	Surgeon (general)		22,811		2185		10.4 (9.1)		4.16 (1.3)							
	Cardiothoracic surgeon		1954		202		9.6 (7.8)		4.54 (1.0)							
	Cosmetic/plastics		13,226		793		16.7 (16.1)		4.05 (1.3)							
	Otolaryngology		10,064		736		13.6 (11.4)		3.87 (1.4)							
	Neurosurgeon		4686		363		12.9 (10.7)		4.17 (1.3)							
	Ophthalmologist		12,419		1305		9.5 (8.5)		3.90 (1.3)							
	Orthopedics/sport		22,492		1770		12.7 (10.0)		3.91 (1.4)							
	Bariatric/weight loss		346		26		13.3 (30.5)		4.05 (1.4)							
	Urologist		9655		772		12.5 (9.5)		4.00 (1.3)							
	Vascular surgeon		392		83		4.7 (5.0)		4.13 (1.4)							
Neurology				9504		944		10.1 (9.5)		3.59 (1.4)		829.2 (83.1)		1.6		1.2
Pediatrics				20,751		1767		11.7 (10.7)		4.1 (1.2)		2488.0 (508.9)		3.1		3.8
Radiology				1005		330		3.0 (2.8)		4.18 (1.3)		2153.7 (216.2)		0.6		3.3
Emergency/critical care^c^				7716		1404		5.5 (5.4)		3.81 (1.5)		860.2 (260.0)		2.4		1.1
**Psychiatry**				18,036		2853		6.3 (6.4)		3.55 (1.5)		4218.9 (550.5)		5.0		6.3
	Psychiatry (general)		17,695		2784		6.4 (6.4)		3.55 (1.5)							
	Addiction medicine		341		69		4.9 (5.6)		3.49 (1.5)							
Dermatology				11,587		705		16.4 (14.9)		3.53 (1.4)		540.0 (27.0)		1.2		0.8
Pathology				57		13		4.4 (8.1)		4.18 (1.2)		1271.4 (100.1)		<0.01		1.9
Genetics				68		26		2.6 (4.9)		4.61 (0.8)		75.8 (12.6)		<0.01		<0.01
Physical medicine/rehabilitation				2517		344		7.3 (8.3)		3.71 (1.5)		372.3 (41.2)		0.6		0.6
Totals/means (SD)				640,603		57,412		11.2 (10.1)		3.9 (1.3)		66,026.1 (5748.2)				

^a^For each specialty, number of unique physicians rated online per total number of unique physicians rated online, expressed as a percent.

^b^For each CIHI physician specialty, mean annual number of physicians per mean total number of annual physicians (2005-2013) expressed as a percent.

^c^Emergency/critical care, as a grouped CIHI specialty, was only available for the years 2009-2013; therefore, annual means were calculated over 5 years only for this specialty.

**Figure 2:**
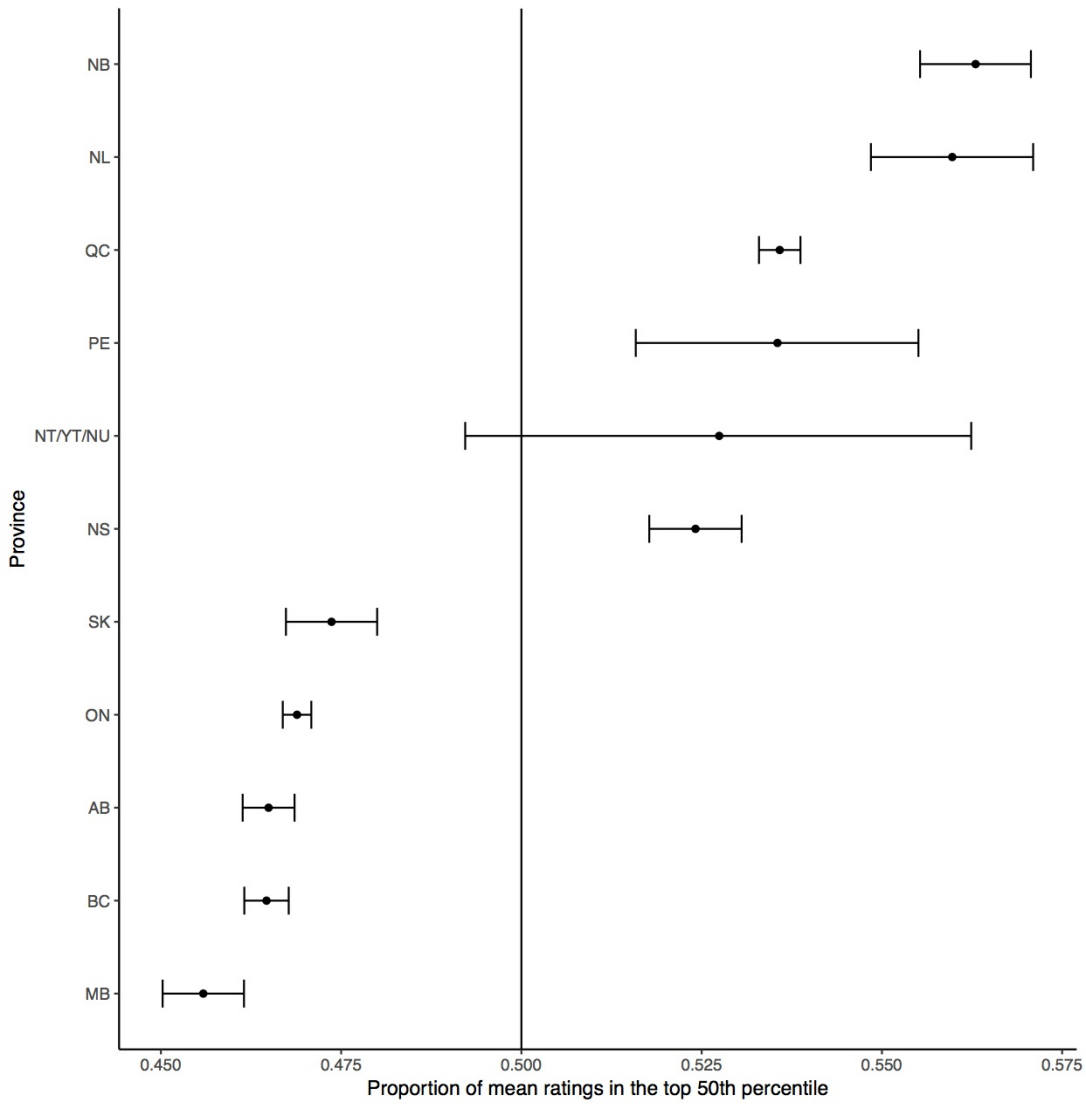
Proportion of mean ratings, by province, in the top 50th percentile of all rated physicians (2005-2013) with 95% confidence intervals depicted for each proportion. NB: New Brunswick; NL: Newfoundland and Labrador; QC: Quebec: PE: Prince Edward Island; NT/YT/NU: Northwest Territories/Yukon/Nunavut; NS: Nova Scotia: SK: Saskatchewan; ON: Ontario; BC: British Columbia; AB: Alberta: MB: Manitoba.

Specifically, physicians practicing in New Brunswick (56.3%, *P*<.001), Newfoundland (56.0%, *P*<.001), Quebec (53.6%, *P*<.001), Prince Edward Island (53.6%, *P*<.001), the Northwest Territories/Yukon/Nunavut (52.7%, *P*=.13), and Nova Scotia (52.7%, *P*<.001) were more likely to be rated greater than 4.5, whereas those practicing in Saskatchewan (46.4%, *P*<.001), Ontario (46.9%, *P*<.001), British Columbia (46.5%, *P*<.001), Alberta (46.5%, *P*<.001), and Manitoba (45.6%, *P*<.001) were likely to be rated 4.5 or lower (Figure 2; Multimedia Appendix 1).

### Differences in Online Ratings for Year of Rating

**Figure 3:**
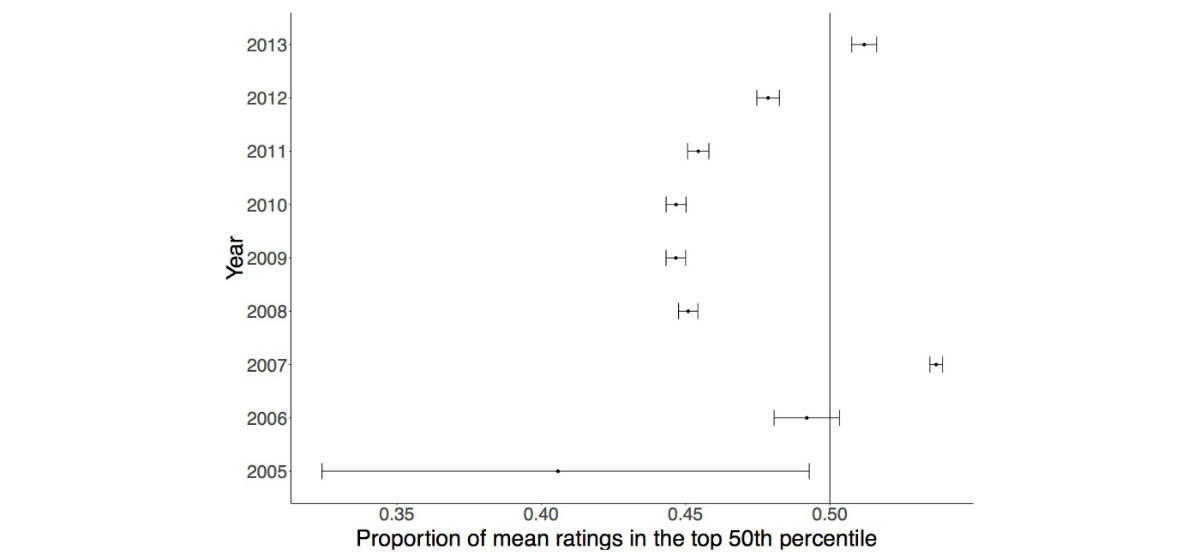
Proportion of mean ratings, per year, in the top 50th percentile of all rated physicians (2005-2013) with 95% confidence intervals depicted for each proportion.

During our study period, there were 640,603 total individual ratings of 27,181 physicians. Over time, the total number of ratings continued to increase; however, we found some important differences in the number of additional new ratings per year (Table 2). In 2005, when the website was still new in Canada, there were only 138 ratings. However, in 2007, 200,650 new ratings were posted before slowly tapering down each subsequent year until 2013, when there were 51,800 new ratings. The year 2007 was also notable in that the mean number of ratings per physician was highest at 5.74 (SD 5.28) before settling at 1 to 3 ratings per physician. In terms of quality of ratings, from 2005 to 2013, physicians were more likely to be rated above the median if rated more recently (ie, in 2013; upper 50th percentile proportion 0.512, *P*<.001), and the likelihood of favorable ratings increased over time. There were two years (2013 and 2007) when quality of ratings were especially high, whereas for the remaining years the proportion of ratings greater than 4.5 was significantly less than 50% (Figure 3; Multimedia Appendix 1).

**Table 2:** Number of ratings, number of physicians, mean ratings per physician, mean overall rating, and additional ratings per year of all physicians rated on RateMDs by province and by year of rating (2005-2013).

Category		Ratings, n	Physicians, n	Ratings per physician, mean (SD)	Mean overall rating, mean (SD)	Additional ratings per year
**Province**						
	New Brunswick	16,128	1447	11.15 (8.9)	4.03 (1.29)	—
	Newfoundland	7564	893	8.47 (7.2)	4.09 (1.23)	—
	Prince Edward Island	2534	242	10.47 (8.0)	4.00 (1.29)	—
	Quebec	116,041	13,460	8.62 (8.5)	4.04 (1.28)	—
	Northwest Territories/Yukon/Nunavut	802	126	6.37 (6.0)	3.94 (1.34)	—
	Nova Scotia	23,482	1992	11.79 (9.5)	3.99 (1.28)	—
	Saskatchewan	24,093	1880	12.82 (11.7)	3.84 (1.34)	—
	Ontario	244,635	20,740	11.80 (10.4)	3.86 (1.33)	—
	Alberta	74,077	5968	12.41 (11.1)	3.86 (1.32)	—
	British Columbia	101,152	8398	12.04 (9.9)	3.87 (1.31)	—
	Manitoba	30,096	2266	13.28 (12.4)	3.80 (1.32)	—
**Year of rating**						
	2005	138	132	1.05 (0.2)	3.75 (1.23)	138
	2006	7726	4280	1.77 (1.4)	3.91 (1.25)	7588
	2007	208,376	34,961	5.74 (5.3)	4.03 (1.23)	200,650
	2008	293,001	28,945	2.92 (2.3)	3.86 (1.32)	84,625
	2009	375,670	28,885	2.86 (2.2)	3.84 (1.33)	82,669
	2010	454,689	30,384	2.60 (2.0)	3.82 (1.35)	79,019
	2011	525,815	30,079	2.36 (1.8)	3.84 (1.33)	71,126
	2012	588,803	29,436	2.14 (1.7)	3.84 (1.37)	62,988
	2013	640,603	27,181	1.91 (1.3)	3.86 (1.42)	51,800

## Discussion

Using national-level data over a nearly 8-year period from the country’s largest physician-rating website, we found that 57,412 unique physicians are rated online and that, overall, ratings are positive. We found differences in ratings with respect to physician specialty, geographic practice location, and year.

To our knowledge, this study is the first to describe the landscape of physician ratings in Canada. This adds to the body of national-level literature on physician-ratings websites in China, Germany, and the United States [6,10,12-14]. Previous studies have focused on either specific specialties or had shorter study periods [20-26]. Overall, our findings are in keeping with previous work that physician ratings are typically positive [6,12-14,17-19,28].

We found that certain specialties (eg, cardiac surgeons and nephrologists) were more likely to be rated in the top 50th percentile of all rated physicians, whereas others (eg, sleep disorder specialists, dermatologists, and addiction medicine specialists) were less likely to be rated as favorably. A variety of physician and patient factors may contribute to such differences. This may be due to differences in patient population as well as differences in patient expectations. For example, surviving a surgery may be a relatively straightforward “rateable” aspect for a surgeon; insight into recognizing the milestones for recovery from addiction with frequent relapses may not be as straightforward. In addition, there are likely more complex interactions between preconceived expectations patients have regarding their physician, their perceived performance of that physician, and their resulting satisfaction—as well described by the expectation-disconfirmation theory in the psychology and consumer marketing literature [42].

Our results add additional information and detail to previous work. Quality of ratings have been shown to be similar for physicians in primary care, medical specialties, surgeons and surgical specialties, and obstetrics and gynecology, but significantly differed for a category of “other physicians,” which included radiologists, pathologists, and anesthesiologists [6]. Others have shown that pediatricians and surgeons had more favorable ratings, although others showed that ratings for generalists did not differ either in quantity or quality from those for subspecialists [17].

In addition to quality of ratings, we also looked at frequency of ratings by specialty. Certain specialties (eg, obstetrics, dermatology, and family medicine) were more commonly rated than others (ie, pathology and radiology), which based on their proportion in the national physician population, overall, in keeping with previous work [6,12,14]. One hypothesis is that patient-physician encounters during surgeries and pregnancies may be discrete care episodes that may be more amenable to appraisal. Also, specialties such as family medicine involve direct physician-patient interaction over time; in contrast, patients rarely interact with their pathologist or radiologist, the two least-rated specialties. Patients may also more readily attribute care to (and hence, rate) a single provider in the case of a surgeon, obstetrician, or dermatologist, as opposed to settings such as inpatient internal medicine, where multiple physicians may collaborate.

We also found differences in the likelihood of a positive rating for geographic location. It seems unlikely that physician quality vastly differs regionally, given the national accreditation and continuing education standards. We noted, in general, that east coast and territory provinces were more likely to have ratings greater than the median (4.5) compared with provinces west of Ontario. There may be geographic differences in rater expectations for a variety of reasons; for example, location may give rise to differences in accessibility to medical care. One interesting hypothesis is that when physicians are scarce, consumers may be more appreciative of access to a physician and this may bias their ratings in a more favorable manner. In addition, we looked at economic prosperity indicators such as gross domestic product by province and found that, overall, lower patient satisfaction is found in more economically prosperous provinces (ie, central and western provinces) [43], in contrast to a theory by Grigoroudis et al [44] that posits that higher patient satisfaction may be explained by economic prosperity. Moreover, other sociologic or cultural phenomenon across locations may lead to variable consumer preference, a well-described marketing phenomenon known as *geographic segmentation* [45]. Explanations for such differences are likely multifactorial and remain, as yet, unknown. There is limited research on the variability of online physician ratings with geographic practice location. Gao et al [6] reported that physicians in the southern United States were slightly more likely to be rated favorably than those practicing in the rest of the country. However, others have reported no difference in ratings regarding practice location and city size for certain surgical specialties [20-22].

Finally, we found differences for ratings over time. We suspect that this is due to patient factors, rather than physician factors, because we would not expect physician quality to fluctuate dramatically from year to year, and the survey instrument was consistent throughout the time period. Of note, RateMDs was founded in 2004 in the United States and, by 2005, online physician websites were still new in Canada (138 ratings in 2005). By 2007, popularity peaked at 200,650 ratings before stabilizing and decreasing by 2013. It is challenging to explain this phenomenon. It may be that in 2007, online physician ratings finally received public attention, resulting in a flood of “early adopters,” which subsequently waned. There was sufficient popularity of such websites and several prominent nationwide media articles in 2007 that physicians became concerned about their use. One article in a popular national news source reported the Canadian Medical Association’s displeasure at such sites and, in particular, warned of the potential for libel [46-48]. However, our findings suggest that these early users were actually more likely to post favorable ratings. This may be plausible, if only because physician-rating website users in general tend to have more positive views toward the Internet, despite no differences in total quantity of Internet usage from the general population [16]. This is in keeping with our finding that the likelihood of a positive rating was highest in 2007.

Since 2007, ratings stabilized and even decreased in absolute number through to 2013. This finding differs from US data, which shows physician-rating website usage rapidly increasing, although the study period in question spans a 5-year period that ends before this study making comparisons problematic [6]. Based on user traffic to competing physician websites in Canada, it does not appear that increasing popularity of competing websites is the explanation. Compared to the United States, in Canada there is comparatively less consumer choice in physician selection because the avenue to seek subspecialty consultation is via one’s primary care physician rather than self-referral. This may, in turn, be driving a decrease in the popularity of physician-rating websites. This hypothesis has been used to explain the use of physician websites in England; although increasing over time as well, they have demonstrated a more gradual, stable rise in popularity compared to the rapidly accelerating US growth [39].

We acknowledge several limitations to our work. First, although our dataset spans nearly an 8-year period, we are missing data from a period of 3 months (ie, October-December 2013 to complete calendar year 2013). However, we feel a national database of greater than 57,000 physicians for nearly an 8-year period is sufficient to elucidate broad trends. Second, online physician-ratings data may not be generalizable. Rating website users likely differ from the general population by virtue of computer access and ability, and by their inclination to post ratings [30]. In addition, because all physicians are entered into the website by raters, it is possible that a physician may have two unique profiles. This database was deidentified; therefore, we were unable to ensure that duplicate profiles were corrected. Moreover, ratings are anonymously posted, so it is possible that fraudulent ratings exist; however, the website has quality control mechanisms in place to circumvent multiple fraudulent ratings (eg, deleting multiple reviews from a single Web address). Third, we could not control for the possibility that online ratings may, themselves, influence future ratings. For example, when a user logs onto the website to post a rating, their original inclination may be influenced by what has previously been published. Overall, these are issues that are germane to most physician-ratings websites and, on balance, we do not feel these limitations would significantly alter our observations, greatly affect broad trends of average ratings and regional differences, nor affect our conclusions.

This study provides new national-level information on the nature of online physician ratings, particularly regarding specialty, geographic practice location, and changes over time. It remains to be seen whether such trends will continue. The utility of online ratings for ascertaining and evaluating physician quality is still in question—and we would argue that before undertaking these larger questions, a better understanding of the scope and breadth of online physician ratings is required. Our study has shown important differences in how physicians are rated based on a physician’s specialty, practice location, and the year in which the physician is rated. Further studies endeavor to better understand the scope, breadth, and utility of online physician ratings; in the meantime, what we do know is that such websites reflect the unsolicited views of the health care consumer and, as such, remain a valuable data source of the patient experience.

## Appendix

Multimedia Appendix 1 Proportion of ratings in top 50th percentile.

## Abbreviations

CIHI: Canadian Institute of Health Information
