# Supplementary material for: Scope, Breadth, and Differences in Online Physician Ratings Related to Geography, Specialty, and Year: Observational Retrospective Study
Source: J Med Internet Res. 2018 Mar 7;20(3):e76. doi: 10.2196/jmir.7475 (PMC5863010; doi:10.2196/jmir.7475)
Supplement: Multimedia Appendix 1 [file jmir_v20i3e76_app1.pdf]

	Proportion of ratings in top 50th percentile	95% CI		p-value
**Specialty**				
Family Medicine	0.463	0.461	0.464	<0.001
Allergy/Immunologist	0.478	0.459	0.497	0.022
Cardiologist	0.632	0.621	0.642	<0.001
Colorectal/proctologist	0.583	0.526	0.638	0.004
Gastroenterologist	0.513	0.503	0.523	0.013
Endocrinologist	0.454	0.441	0.467	<0.001
Reproductive endocrinologist	0.487	0.460	0.513	0.326
Geriatrician	0.521	0.482	0.559	0.300
Infectious Disease	0.554	0.524	0.584	<0.001
Internist	0.525	0.513	0.537	<0.001
Nephrologist	0.692	0.670	0.712	<0.001
Oncology/Hematologist	0.582	0.570	0.594	<0.001
Pulmonologist	0.591	0.571	0.611	<0.001
Rheumatologist	0.468	0.455	0.481	<0.001
Sleep disorders	0.450	0.399	0.502	0.062
Anesthesia	0.616	0.597	0.635	<0.001
Obstetrics and Gynecology	0.468	0.463	0.473	<0.001
Surgeon (general)	0.621	0.615	0.627	<0.001
Cardiothoracic surgeon	0.781	0.762	0.799	<0.001
Cosmetic/Plastics	0.583	0.574	0.591	<0.001
Otolaryngology (ENT)	0.498	0.488	0.508	0.727
Neurosurgeon	0.629	0.615	0.627	<0.001
Ophthalmologist	0.482	0.473	0.491	<0.001
Orthopedics/Sport	0.516	0.509	0.522	<0.001
Bariatric/Weight Loss	0.587	0.533	0.639	0.002
Urology	0.544	0.534	0.554	<0.001
Vascular surgeon	0.651	0.601	0.697	<0.001
Neurology	0.421	0.411	0.431	<0.001
Pediatrics	0.547	0.541	0.554	<0.001
Radiology	0.653	0.622	0.682	<0.001
Emergency/Critical Care	0.538	0.527	0.549	<0.002
Psychiatry	0.422	0.415	0.430	<0.001
Addiction Medicine	0.358	0.307	0.411	<0.001
Dermatology	0.370	0.361	0.379	<0.001
Pathology	0.509	0.374	0.642	1.000
Genetics	0.735	0.612	0.832	<0.001
Physical Medicine/Rehabilitation	0.486	0.467	0.506	0.175
**Geographic practice location**				
New Brunswick	0.563	0.555	0.571	<0.001
Newfoundland	0.560	0.548	0.571	<0.001
Prince Edward Island	0.536	0.516	0.555	<0.001
Quebec	0.536	0.533	0.539	<0.001
Northwest Territories/Yukon/Nunavut	0.527	0.492	0.562	0.129
Nova Scotia	0.524	0.518	0.531	<0.001

Saskatchewan	0.474	0.467	0.480	<0.001
Ontario	0.469	0.467	0.471	<0.001
Alberta	0.465	0.461	0.469	<0.001
British Columbia	0.465	0.461	0.468	<0.001
Manitoba	0.456	0.450	0.462	<0.001
**Year of rating**				
2005	0.406	0.324	0.493	0.033
2006	0.492	0.481	0.503	0.165
2007	0.537	0.535	0.539	<0.001
2008	0.451	0.448	0.454	<0.001
2009	0.447	0.443	0.450	<0.001
2010	0.447	0.443	0.450	<0.001
2011	0.454	0.451	0.458	<0.001
2012	0.479	0.475	0.482	<0.001
2013	0.512	0.508	0.516	<0.001
